# Association between Influenza A Virus Infection and Pigs Subpopulations in Endemically Infected Breeding Herds

**DOI:** 10.1371/journal.pone.0129213

**Published:** 2015-06-15

**Authors:** Andres Diaz, Andres Perez, Srinand Sreevatsan, Peter Davies, Marie Culhane, Montserrat Torremorell

**Affiliations:** Department of Veterinary Population Medicine, College of Veterinary Medicine, University of Minnesota, Saint Paul, Minnesota, United States of America; University of Georgia, UNITED STATES

## Abstract

Influenza A viruses (IAVs) are distributed worldwide in birds, pigs and humans, and cause important endemic disease affecting hosts in all countries. Although pigs play a key role in the ecology of IAVs, the epidemiology of IAVs within swine herds is poorly understood. In this longitudinal study we describe the prevalence of IAVs infection in three subpopulations of pigs in 5 breeding herds in the Midwestern USA. Each herd was sampled monthly for a year and, at each visit, 30 individual nasal swabs were collected from the three subpopulations, namely, a) replacement females, resident on-farm for less than 4 weeks (new gilts), b) replacement females, resident on-farm for more than 4 weeks (gilts), and c) neonatal pigs less than 21 days of age (piglets). Real time reverse transcriptase polymerase chain reaction (RRT-PCR) was used to detect IAVs, and the association between IAVs infection and pig subpopulation was measured using a mixed logistic regression model. Nasal swabs (n = 4,190) were collected from 141 groups of pigs. At least, one IAV-positive nasal swab was found in 19.9% (n = 28) of the sampled groups, and 7.7% (n = 324) of all nasal swabs tested positive. After adjusting by annual quarter and sampling event, the odds of testing IAV positive were 7.9 (95% CI 1.4, 43.9) and 4.4 (95% CI 1.1, 17.1) times higher in groups of new gilts and piglets compared to groups of gilts, respectively. Results indicate that new gilts and piglets had higher odds of testing IAV positive than gilts in swine breeding herds and that season influences IAV infection in pigs. Based on these findings, we recommend that IAV control strategies be aimed at preventing infection before gilts are introduced into the farm, and in pigs prior to weaning.

## Introduction

Influenza A viruses (IAVs) are Orthomixoviruses able to infect many animal species including birds, pigs and humans [1]. The segmented genome of IAVs allows the exchange of gene segments between IAVs during infection and replication [2] facilitating the emergence of novel IAV reassortants with pandemic potential. The 2009 pandemic IAV contained genes from swine IAVs circulating in North America and Eurasia [3] and highlighted the importance of pigs in the ecology of IAVs among species.

Influenza-like disease was first reported in pigs in 1918 at the time of the human Spanish flu pandemic and the virus was first isolated from pigs in 1930 [4, 5]. Currently, IAV infections occur worldwide and are considered endemic in swine populations [4]. In the US, IAVs have been present in pigs for many decades and several serological surveys conducted since the 1970’s have consistently demonstrated that IAVs are ubiquitous in swine [6–8]. IAVs remained genetically stable in pigs in the US with minimal or undetected viral evolution until 1998 when H3N2 reassortant viruses of swine, human and avian origin were detected in pigs [5]. Subsequently new strains, new subtypes, and multiple reassortant viruses have been identified in pigs in North America [9, 10]. There is evidence that multiple human introductions of IAVs, including the 2009 H1N1 pandemic virus, into the pool of IAVs circulating in the US have greatly contributed to the increase of genetic diversity of the virus [10, 11]. The contemporary swine farming methods and live animal movements between farms and geographical regions increase IAV diversity [12, 13] and make IAVs harder to control in swine populations. However, limited information is available about the frequency of IAV introduction and its maintenance within swine herds.

IAV infections are common in the US Midwest, and herds can test positive year around with diverse viruses, regardless of the presence or absence of clinical signs and vaccination status [14]. Although influenza infections in individual pigs are of short duration (5–7 days), IAV infections of herds can be prolonged (weeks or months). When population dynamics, and extended IAVs circulation in pig farms are taken in consideration, the likelihood of reassortment increases [15, 16]. Furthermore the introduction of IAV strains of human origin broadens virus diversity in pigs [17] and this diversity is accentuated by the frequent movement of weaned pigs into swine-dense areas [13].

The contemporary US swine industry is mostly organized into multi-site production systems. These systems have defined production stage facilities located in different geographical sites. Breeding, gestation and farrowing takes place in breed-to-wean (breeding) herds. At approximately 21 days of age, piglets are weaned and transported to a nursery or a wean-to-finish site where pigs are raised until 10 weeks of age or to market-age respectively. Breeding herds house replacement animals (gilts), adult females (sows), and piglets. Except on breeding farms where farrowing occurs in batches, typically piglets are born daily, with weekly births totaling about 40% of the resident adult female population. Adult females are replaced at a yearly rate of 45 to 55% [18], and replacement gilts are regularly introduced on schedules that range from 1 to 10 weeks across farms. As a result, breeding populations have high rates of turnover, and associated fluctuations in herd’s susceptibility to IAV infections. Previous studies have shown that it is not easy to recover IAVs from sows [15, 19, 20]. However, the dynamics of IAV infection among other subpopulations (gilts and suckling piglets) may influence the maintenance of IAVs in the breeding herd or contribute to the emergence of novel strains.

In this study, we aimed to define patterns of IAV infection in the most labile subpopulations on 5 IAV-infected breeding farms, with the goal of understanding the relative importance of these subpopulations as sources of new IAV infections in the breeding herd. This information is necessary to design targeted strategies to control IAV infection within and between swine herds, and to minimize the risk of IAV infections of swine origin to people.

## Materials and Methods

### Ethics statement

Protocols and procedures followed throughout the study were approved by the University of Minnesota Institutional Animal Care and Use Committee (IACUC 1207B17281), and the Institutional Biosafety Committee (IBC 1208H18341).

### Study design and sample collection

**Fig 1:**
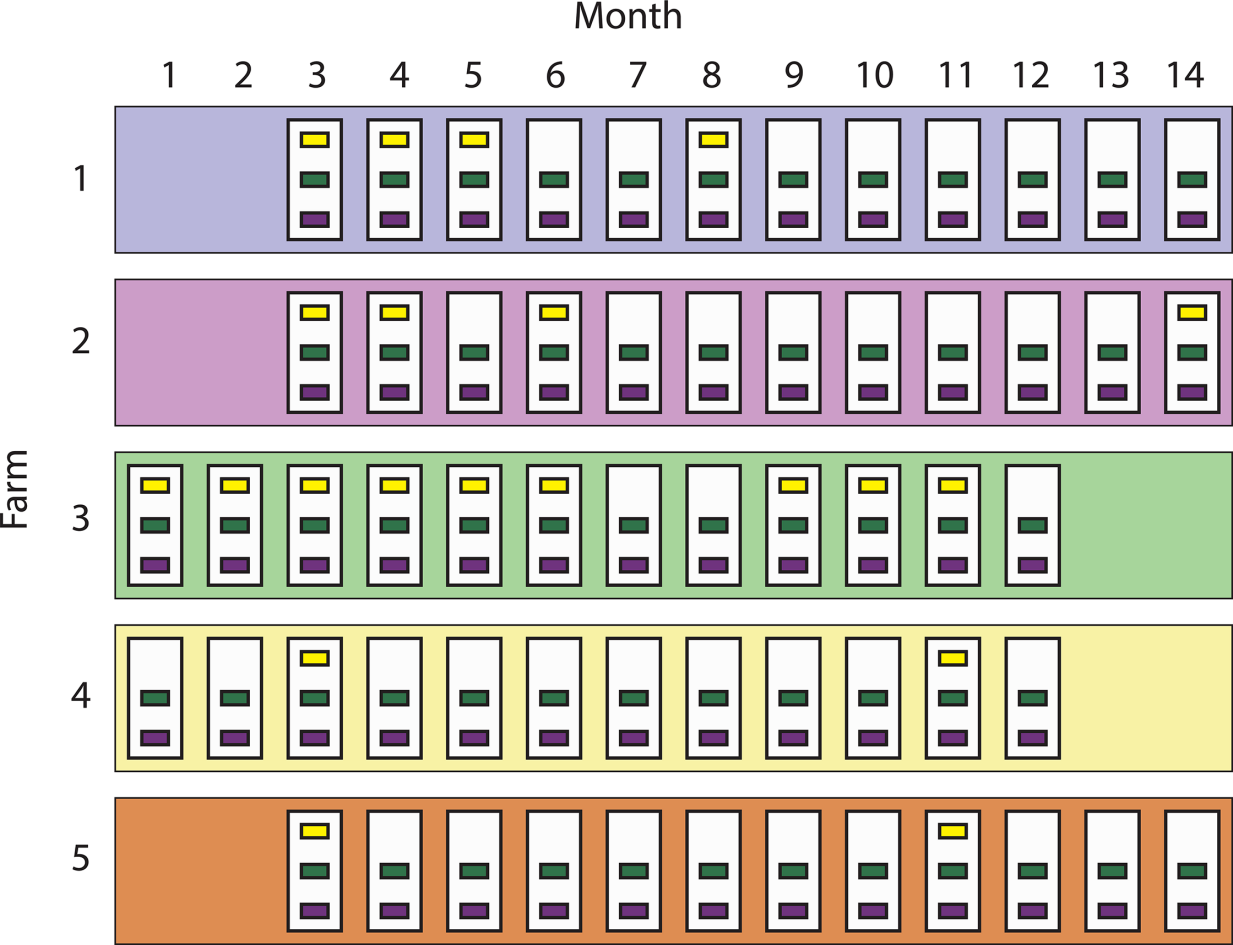
Study design. Five swine-breeding herds (largest rectangles, 1 to 5) in the Midwest were sampled between November 2011 (month 1) and December 2012 (month 14). White rectangles represent sampling visits (12 per farm), and the smallest rectangles (n = 141) indicate the groups of pigs sampled. Groups are colored based on pig subpopulation: yellow (new gilts, n = 21), green (gilts, n = 60), and purple (piglets, n = 60). Missing group-rectangles indicate that there were no new gilts on that visit. Groups were assumed nested within sampling events, and sampling events were assumed nested within farms.

Five commercial pig-breeding herds (Farms 1 to 5) located in the Midwestern USA were conveniently selected for this study. Selection criteria included breed-to-wean herds with: a) confirmed IAV infection by real time reverse transcriptase polymerase chain reaction (RRT-PCR) within the past year, b) presence of an on-site gilt development unit (GDU), and c) introduction of external replacement animals into a defined isolation area. Each farm was visited monthly for 12 months and the overall sampling period spanned from November 2011 to December 2012. At each visit, three pig subpopulations were sampled: a) replacement females, resident on-farm for less than 4 weeks (new gilts), b) replacement females, resident on-farm for more than 4 weeks (gilts), and c) neonatal pigs less than 21 days of age (piglets). Adult animals (sows) were not included since prior studies consistently reported a low probability of influenza detection in this subpopulation [15, 19, 20]. Due to schedules for delivery of replacement females, eligible populations of new gilts were only present at 21 of the 60 sampling events. A diagram of the study design can be seen in Fig 1. Information on management and control practices for IAV was recorded during the last visit.

Thirty pigs were selected from each pig subpopulation (new gilts, gilts and piglets) and sampled individually using a nasal swab (BBL CultureSwab, Becton Dickinson and Company, USA). Sample size (n = 30) was estimated based on a 95% confidence to detect at least 1 positive sample if prevalence was 10% or higher at the subpopulation level. After sample collection, nasal swabs were refrigerated and transported to the laboratory on the manufacturer’s transport media. At the laboratory, swabs were placed on 1.8 ml viral transport media (Dulbecco’s Modified Eagle Medium (DMEM) plus 5% antibiotic-antimycotic, Gibco Life technologies, USA) vortexed for 10 seconds, alliquoted and stored at -80°C until testing.

### Influenza A virus detection and subtyping

Nasal swabs were first screened for IAV by RRT-PCR on pools of three samples. If a pool tested positive then the samples comprising the pool were tested individually. All samples in a negative pool were considered negative. Viral RNA was eluted using 50 μl of each sample into 50 μl elution buffer using MagMax virus RNA isolation kit (Ambion, USA). Primers targeting the matrix (M) gene and AgPath-ID One-Step RT-PCR reagent kit (Ambion, Life technologies, USA) were used to detect IAV [21, 22]. PCR mix containing 5 μl RNA, 12.5 μl 2X buffer, 1.0 μl 25X enzyme mix, 1.67 μl detection enhancer, 5 pmol of each primer and 1.5 pmol of probe was run on a LightCycler 480 system (Hoffmann-La Roche, Switzerland) at 45°C for 10 min, followed by 95°C for 10 min, and 45 cycles at 94°C for 1 sec and 60°C for 30 sec. Fluorescence was recorded at 60°C and a sample was considered positive if the cycle threshold (CT) was lower than 40. Positive samples with a CT value of 35 or lower were used for IAV virus isolation on Madin–Darby Canine Kidney (MDCK) cells [23] and each IAV isolate was subtyped based on the hemagglutinin and neuraminidase combination [24].

### Data analysis

Data collected in the survey was summarized by farm and three independent variables were taken into consideration for statistical analysis: a) pig subpopulation, b) farm, and c) annual quarter (1^st^ to 4^th^). The association between IAV detection and subpopulation was analyzed at the group level where a group was defined as the subpopulation of pigs that was sampled during a given visit and considered positive if one or more swabs within the group tested positive to IAV. A Pearson’s Chi square or Fisher’s exact test was used to compare the frequency of IAV positive and negative outcomes by subpopulations (new gilts, gilts and piglets), farms (1 to 5), and annual quarter. Using gilts as the reference group, an unconditional logistic regression model was used to measure the crude association between the outcome and pig subpopulations, farm and annual quarter. Finally, fixed and mixed logistic regression models were compared to estimate the associations between IAV infection and subpopulation adjusting by farm, annual quarter, and sampling event. Subpopulation and annual quarter were included as fixed effects in the models, and clustering variables (e.g. farm and farm visit) were included as random effects. Given the study design (Fig 1) we assumed that samples clustered by sampling visit, farm, and annual quarter. We also assumed that pig subpopulations were nested within sampling visits, and that sampling visits were nested within farm. A Wald Chi-square was used to test the significance of individual coefficients within each model and a likelihood ratio Chi square test was used to compare hierarchical models. Statistical significance was assumed at p values lower than 0.05 and the model with the lowest Akaike information criterion (AIC) value was selected as the final model. All data analysis and graphics were performed using R 3.1.0 (The R Foundation for statistical Computing, www.R-project.rog) and packages installed included lattice [25], gmodels, car, aod [26], ggplot [27], lme4 [28] and psych [29].

## Results

**Fig 2:**
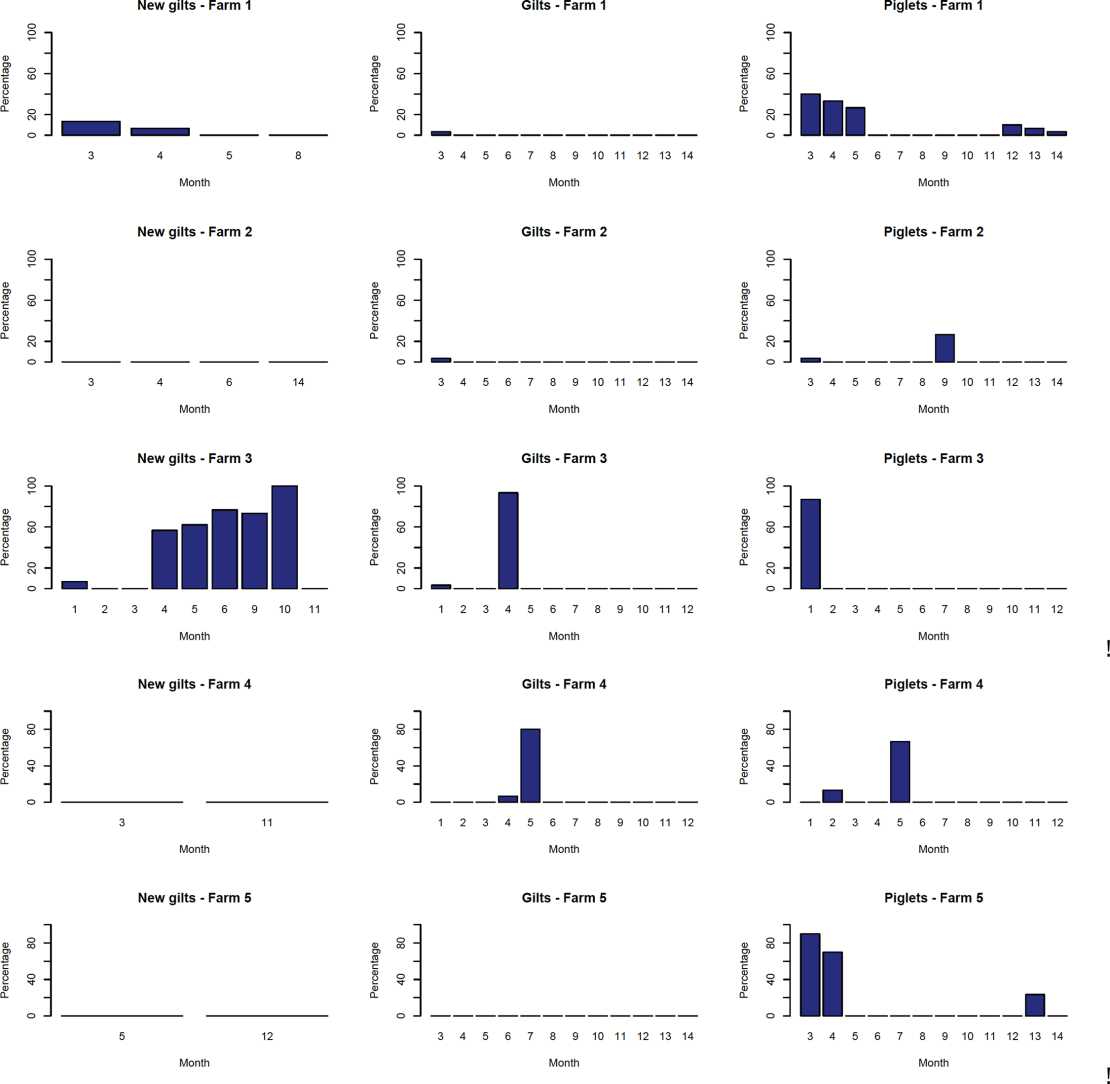
Percentage of influenza A virus positive samples distributed by farm, subpopulation and month.

Between November 2011 and December 2012, 4190 individual nasal swabs were collected from 141 groups of pigs in the 5 pig-breeding herds. Farm demographics are summarized in Table 1. Among all groups sampled, 60 (42.5%), 60 (42.5%) and 21 (15%) were groups of piglets, gilts and new gilts respectively. Swabs were collected from 1796 (43%) piglets, 1768 (42%) gilts and 626 (15%) new gilts. Although all farms and subpopulations under study tested positive to IAV at least once, only 28 groups (19.9%) and 324 swabs (7.7%) were positive for IAV. Piglets tested positive at least once in all farms, new gilts only tested positive in farms 1 and 3, and all gilts tested negative in farm 5 (Fig 2). One hundred and twenty four IAVs isolates were recovered and 123 of them were successfully subtyped. Subtypes H1N1, H1N2 and H3N2 were identified and more than one IAV subtype was isolated in all farms over time.

**Table 1:** Descriptive farm demographics.

Farm	1	2	3	4	5
State	South Dakota	Minnesota	Iowa	Minnesota	Minnesota
Date when sampling started	Jan-12	Jan-12	Nov-11	Nov-11	Jan-12
Sow inventory	3420	2343	3249	1000	3000
Sow vaccination against IAV	No	Yes	Yes	No	No
Average age of piglets at weaning (days)	19	20	20	18	21
Frequency of gilt introduction (weeks)	5	5	5	20	5
Average age of new gilts at arrival at the farm (weeks)	21	3	3	16^1^	22
Vaccination of gilts after arrival	No	Yes	No	Yes	Yes
Respiratory clinical disease in piglets	Yes	Yes	No	Yes	No
Respiratory clinical disease in new gilts	No	No	Yes	No	Yes

^1^Age range for new gilts at arrival in farm four was between 8 and 24 weeks. For all other farms it was one week.

No positive samples were found in May, June or September 2012 and the univariate analyses indicated that IAV infection was strongly associated (p<0.01) with pig subpopulation and annual quarter (Table 2). The crude odds of IAV infection in groups were higher for piglets and new gilts compared to groups of gilts, and lower for groups of pigs sampled during the second annual quarter (April, May, June) compared to any other quarter of the year (Table 3).

**Table 2:** Number (%) of pig groups positive to influenza A virus by RRT-PCR distributed by subpopulation, farm, and annual quarter.

Variable		Positives (%)	Negatives (%)	Total
Subpopulation (p<0.01)	New gilts	8 (38.1)	13 (61.9)	21
	Gilts	6 (10.0)	54 (90.0)	60
	Piglets	14 (23.3)	46 (76.7)	60
	Total	28 (19.9)	113 (80.1)	141
Farm (p = 0.17)	1	9 (32.1)	19 (67.9)	28
	2	3 (10.7)	25 (89.3)	28
	3	9 (27.3)	24 (72.7)	33
	4	4 (15.4)	22 (84.6)	26
	5	3 (11.5)	23 (88.5)	26
	Total	28 (19.9)	113 (80.1)	141
Annual quarter (p<0.001)	1^st^	16 (40.0)	24 (60.0)	40
	2^nd^	1 (3.0)	32 (97.0)	33
	3^rd^	3 (8.8)	31 (91.2)	34
	4^th^	8 (23.5)	26 (76.5)	34
	Total	28 (19.9)	113 (80.1)	141

A group was considered positive if at least one swab within the group was IAV RRT-PCR positive. The proportion of positive vs. negative outcomes by subpopulation, farm, and annual quarter was compared using a Pearson chi-square test (or Fisher’s exact if needed) and the p-value for each comparison is shown.

**Table 3:** Results from the univariate analysis.

Variable	Group	OR (95% CI)
Subpopulation	Gilts	-
	New gilts	5.5 (1.6, 19.6)***
	Piglets	2.7 (1.01, 8.3) **
Annual quarter	2^nd^. Apr, May, Jun	-
	3^rd^. Jul, Aug, Sep	3.1 (0.4, 64.5)
	4^th^. Oct, Nov, Dec	9.8 (1.7, 188.8)*
	1^st^. Jan, Feb, March	21.3 (3.9, 398.4)**

The crude association between IAV detection and subpopulation or annual quarter was measured through odds ratios. A group was considered positive if one or more swabs within the group tested positive to IAV by RRT-PCR. The first group for each variable of interest was used as the reference group.

*p<0.05

**p<0.01

***p<0.001

The final multivariate model indicated that, after adjusting by annual quarter and sampling visit, the odds of IAV infection were higher in groups of new gilts (OR = 7.9 95% CI: 1.4,43.9) and piglets (OR = 4.4 95% CI: 1.1,17.1) compared to groups of gilts (Table 4). Inclusion of farm to the model was not statistically significant (p>0.05) therefore this variable was excluded from the final model.

**Table 4:** Results from the multivariate analysis (Mixed effects model).

Variable	Group	OR (95% CI)
Subpopulation	Gilts	-
	New gilts	7.9 (1.4, 43.9)*
	Piglets	4.4 (1.1, 17.1) *
Annual quarter	2^nd^. Apr, May, Jun	-
	3^rd^. Jul, Aug, Sep	3.5 (0.2, 54.9)
	4^th^. Oct, Nov, Dec	16.1 (1.1, 234.7) *
	1^st^. Jan, Feb, March	43.9 (2.8, 686.8)**

While subpopulation and annual quarter were included as fix effects, sampling visit was included as random effect.

*p<0.05

**p<0.01

***p<0.001

## Discussion

To advance our understanding of IAV epidemiology in swine breeding herds, we combined frequent sampling and PCR-based methods to define patterns of active IAV infections among pig subpopulations present in these herds. We found that replacement animals resident on-farm for less than 4 weeks (new gilts) and pigs less than 21 days of age (piglets) had higher odds of testing positive to IAVs compared to replacement animals resident on-farm for more than 4 weeks (gilts). Therefore new gilts and piglets may represent the most epidemiologically significant reservoirs for IAVs in swine breeding herds. Sows were not included in our study because they have been found to have a low probability of influenza positivity in endemically affected herds [15, 19, 20]. Our results also indicate that there was a strong association between IAV infection and annual quarter and that this association was still statistically significant after controlling for the subpopulation effect.

In the USA there are approximately 65 million commercial pigs, of which approximately 6 million are breeding sows. Breeding females are replaced at a 45–55% rate annually (i.e. approximately 3 million gilts are introduced every year into US sow farms to replace existing breeding stock), and each sow gives birth and weans approximately 27 and 24 piglets respectively [18]. For example, in a herd of 1000 sows that weans 25 pigs per sow per year at 3 weeks of age, the expected suckling piglet population is approximately 1440 (larger than the sow population), and around 450 piglets are born each week. The implications for IAVs evolution and emergence of new strains in this population undergoing rapid turnover are not fully understood, but the continual availability of new susceptible hosts with different levels of immunity to IAVs (acquired or maternally derived) may favor emergence of new variants.

Most published studies to date have studied IAV transmission at a broad scale based on the genetic evolution of IAV throughout time and space [11–13, 17], and relatively limited information is available on the epidemiology of IAVs at the herd level. Furthermore, most herd level studies have used serological methods rather than direct detection of the virus by molecular methods [30–32]. Serological results are less definitive and can be difficult to interpret since detected antibodies may reflect maternally derived antibodies or active immunity to IAV infection or vaccination, none of which can be distinguished from the other.

This study demonstrates that in certain breeding herds, new gilts and piglets can be an important reservoir for IAVs in swine populations. New gilts represented animals from an external source and could have been naïve to resident farm viruses or a source of new IAVs to the breeding herd. Upon arrival to a farm, new gilts were commonly kept in a separate room or building (although rarely in complete isolation) to minimize introduction of new diseases for approximately 30 days. After that, gilts were moved into the gilt development unit. We selected 4 weeks as the cut-off to classify gilts (new gilts or gilts) to reflect this industry practice of gilt management. Our results then showed that gilts had lower odds of IAV infection. One possible explanation is that gilts have been able to clear the infection since they have been on site for a longer period of time. IAV infections at the individual animal level are self-limiting and usually last between 5 to 7 days [5]. In contrast, finding IAVs more frequently in new gilts may reflect the introduction of infected animals from the source herds. Although in this study we did not sample the source herds, the likelihood of these herds to be IAV positive is high given the commonality of IAV infections in the Midwest [14]. However, we cannot fully rule out the possibility that new gilts became infected with resident viruses after arrival to the herd. Although new gilts were placed into isolated designated areas and procedures were in place to minimize disease transmission (eg. isolation, vaccination), these areas or procedures might not have been able to fully contain infections within the designated areas. Future studies sampling gilts at arrival to the breeding herd would be required to more precisely estimate the risk of gilts at introducing IAVs into breeding herds. Nevertheless our study identified new gilts as a subpopulation that tested IAV positive more frequently than resident gilts which indicates the need to have specific control programs or protocols to mitigate their risk.

In contrast, neonatal piglets at birth are immunologically naïve and likely acquire IAV infection within the breeding herd. The role of piglets in maintenance and dissemination of IAV infections has been documented before [33]. In this study we support those findings although our study design did not discern whether infections in piglets in consecutive months were due to endemic viruses maintained in this subpopulation, or whether they represented new infections from other subpopulations. Previous studies have shown that different IAV subtypes can co-circulate, be sustained [14] and reassort [16] in swine populations. In this study we also demonstrate that different IAV subtypes can be present simultaneously in breeding herds. Further studies on the complete genome characterization and phylogenetic analysis of the viruses recovered from these subpopulations will be able to assess virus diversity and reassortment within these pig subpopulations. Additionally, our study measured the odds of IAV infection based on the detection of the matrix gene. The matrix gene segment is highly conserved among IAVs. Whether the odds of IAV infection in pig subpopulations varies for different IAV subtypes should also be further investigated.

Time of the year when IAV infection was detected was also associated with IAV infection in our study. In humans, IAV infections have a clear seasonal pattern in temperate regions but the pattern is less defined in the tropics [34]. In swine, IAVs seasonality is still under debate [14, 35, 36]. One recent study in Europe did not detect any seasonal trend of swine IAV [37]. In another study, season (winter) and IAV like-illness in pigs were strongly associated but IAVs detection in pigs and season were not [36]. In our study, we found higher odds of IAV detection in groups of pigs sampled during the first quarter of the year (winter in the northern hemisphere), thus favoring the hypothesis of a seasonal trend of swine IAV. The differences among studies may be due to study design, sample size, and the result of measuring these associations using active or passive surveillance.

We acknowledge that our results do not represent IAV infection dynamics across U.S herds since only a limited number of herds participated in the study and these herds were conveniently selected. In addition, we identified significant variability in the number of IAV infected pigs between sampling events, which may be a reflection of the sample size or number of farms in the study. However, our IAV detection rates are similar to those described in other studies and our sample size for each of the farms is larger than in other studies [38].

In conclusion, our study indicates that there are differences in the odds of IAV infection across different pig subpopulations found in breeding herds, and that IAVs can be found more frequently in new gilts and piglets than in resident gilts. Our results also indicate that there is a strong seasonal component to IAV infection in breeding herds from this study with the first quarter of the year being the period with most positives. Overall our results contribute to better understand IAV transmission in pigs and indicate the need to focus interventions to control IAV infections in new gilts and piglets before weaning.

## Supporting Information

S1 ARRIVE Checklist Animal Research: Reporting of In Vivo Experiments check list. (PDF)
